# Immune Responses Raised in an Experimental Colon Carcinoma Model Following Oral Administration of *Lactobacillus casei*

**DOI:** 10.3390/cancers12020368

**Published:** 2020-02-05

**Authors:** Georgios Aindelis, Angeliki Tiptiri-Kourpeti, Evangeli Lampri, Katerina Spyridopoulou, Eleftheria Lamprianidou, Ioannis Kotsianidis, Petros Ypsilantis, Aglaia Pappa, Katerina Chlichlia

**Affiliations:** 1 Department of Molecular Biology and Genetics, Democritus University of Thrace, University Campus Dragana, 68100 Alexandroupolis, Greece; g.aindelis@gmail.com (G.A.); mbg_tiptiri@yahoo.gr (A.T.-K.); evangeli.lampri@gmail.com (E.L.); aikspiridopoulou@gmail.com (K.S.); apappa@mbg.duth.gr (A.P.); 2 Department of Hematology, School of Medicine, Democritus University of Thrace, University Campus Dragana, 68100 Alexandroupolis, Greece; lamprianidou_el@yahoo.gr (E.L.); ikotsian@med.duth.gr (I.K.); 3 Laboratory of Experimental Surgery and Surgical Research, School of Medicine, Democritus University of Thrace, 68100 Alexandroupolis, Greece; pipsil@med.duth.gr

**Keywords:** probiotics, syngeneic murine colon carcinoma, immune responses, oral administration, *Lactobacillus casei*

## Abstract

The role of dietary probiotic strains on host anti-cancer immune responses against experimental colon carcinoma was investigated. We have previously shown that *Lactobacillus casei* administration led to tumor growth suppression in an experimental colon cancer model. Here, we investigated the underlying immune mechanisms involved in this tumor-growth inhibitory effect. BALB/c mice received daily live lactobacilli *per os* prior to the establishment of a syngeneic subcutaneous CT26 tumor. Tumor volume, cytokine production, T cell differentiation and migration, as well as tumor cell apoptosis were examined to outline potential immunomodulatory effects following *L. casei* oral intake. Probiotic administration in mice resulted in a significant increase in interferon gamma (IFN-γ), Granzyme B and chemokine production in the tumor tissue as well as enhanced CD8^+^ T cell infiltration, accompanied by a suppression of tumor growth. Cytotoxic activity against cancer cells was enhanced in probiotic-fed compared to control mice, as evidenced by the elevation of apoptotic markers, such as cleaved caspase 3 and poly (ADP-ribose) polymerase 1 (PARP1), in tumor tissue. Oral administration of *Lactobacillus casei* induced potent Th1 immune responses and cytotoxic T cell infiltration in the tumor tissue of tumor-bearing mice, resulting in tumor growth inhibition. Thus, the microorganism may hold promise as a novel dietary immunoadjuvant in raising protective anti-cancer immune responses.

## 1. Introduction

Probiotics are defined as live microorganisms that when administered in adequate amount confer a health benefit to the host [1]. Most probiotics are part of a heterologous group known as lactic acid bacteria (LAB) (*Lactobacillus, Enterococcus*) and bacteria of the genus *Bifidobacteria* [2].

A wide variety of disorders have been shown to respond positively to LAB administration, such as intestinal inflammation [3], diarrhea [4], allergic diseases [5] and psychiatric disorders [6]. Recent studies suggest that some of them exhibit immunoregulatory properties, amplifying host defense mechanisms, especially against colon cancer [7]. Activation of specialized anticancer immune responses seems to be part of their repertoire, although how this is achieved has not been fully verified [8].

Various mechanisms have been put forward as potential explanations for the effects exhibited by probiotics. Among them are alterations in the gut microbiota, antagonizing pathogenic strains [9], strengthening of the intestinal mucosal barrier [10], regulation of immune responses [8], direct anti-proliferative and pro-apoptotic effects [11] and induction of immunogenic cell death [12].

Several in vitro experiments have revealed that probiotic bacteria demonstrate cytotoxicity against human cancer cell lines [13,14]. Viable bacteria, metabolites [15] and cell free extracts [16] have been proposed as anti-proliferative agents. The aforementioned components have also been linked with increased interleukin 1 (IL-1) and tumor necrosis factor alpha (TNF-a) production by murine peritoneal macrophages [17] and increase of Th1 cells induced through a highly controlled IL-12 production by immune cells originating from mouse spleen and Peyer’s patches (PP) [18]. The same effect has also been observed in human activated peripheral mononuclear cells [19,20].

In vivo experiments have revealed notable anti-tumor and immunomodulatory effects [21] following lactobacilli administration [22], as well as modulation of intestinal micro flora, an event that affects host immune responses. This activity has been linked to the increase in stimulatory cytokines that favor cytotoxicity [18]. Intranasal administration of *L. casei BL23* [23] resulted in increased natural killer (NK) cell activity in mice challenged with TC-1 cells, and oral administration of *Lactobacillus plantarum* promoted intratumor migration of CD8^+^ and NK cells [24]. Consumption of probiotic bacteria has also been linked with prolonged survival of tumor-bearing mice, an effect associated with increased IL-12 production [25]. The combination of increased IL-12 secretion with interferon gamma (IFN-γ) production and augmentation of the NK cells and CD4^+^ T cells populations has been verified by multiple studies [26,27]. Sivan et al. have reported that a cocktail of lyophilized *Bifidobacteria* species, when administered in mice bearing B16.F10 melanoma, resulted in the development and accumulation of highly effective tumor-specific CD8^+^ cells, which in combination with anti-programmed cell-death ligand 1 (PD-L1) treatment, nearly abolished tumor overgrowth [28].

Administration of lactobacilli has been documented to not only stimulate production of cytotoxic cell development, but also maturation of Th1 helper T cells [26]. Th1 cytokine production has resulted in regulation of complex signaling pathways of differentiation, development and targeted intra-tumor migration of specialized effector immune cell populations, such as CD8^+^ T cells and NK cells [29,30]. Their recruitment results in increased cancer cell apoptosis through cell interactions and secretion of anti-cancer factors, making the host’s immune response against tumorigenic incidents more efficient [31].

As we had previously reported, *Lactobacillus casei* ATCC 393 demonstrates potent anti-proliferative and pro-apoptotic activity *in vitro* as well as significant tumor growth inhibitory effects in a syngeneic CT26 cancer model when orally administered in tumor-bearing BALB/c mice [11]. In this study, we explored the immunomodulatory properties of the probiotic in the production and migration of effector immune cells, as well as their ability to secrete various chemotactic agents and highly active cytotoxic cytokines, like IFN-γ and granzyme B.

## 2. Results

### 2.1. Oral Administration of Live Lactobacillus casei Suppresses Subcutaneous Colon Carcinoma Growth in Mice

**Figure 1:**
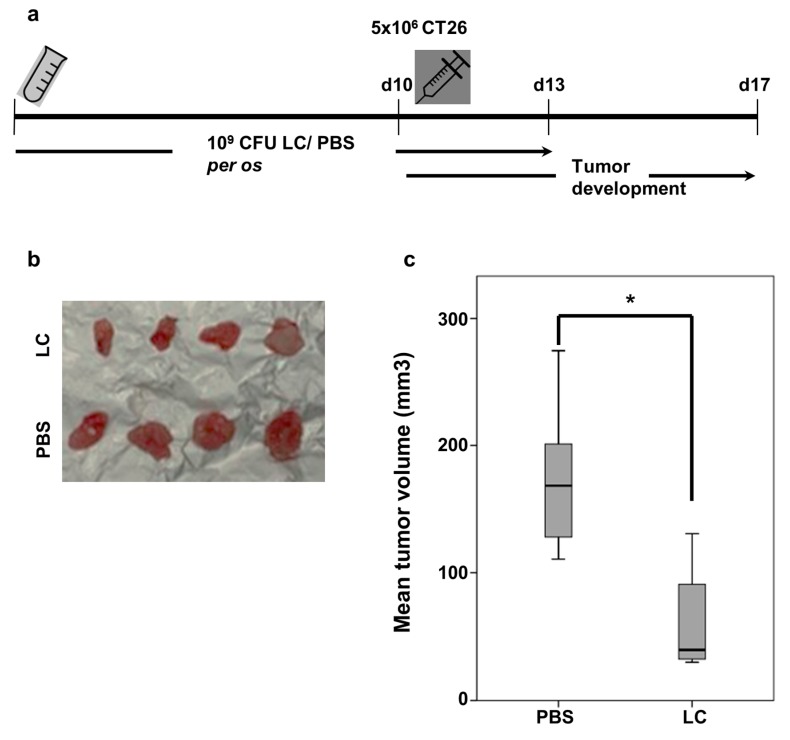
Oral administration of *Lactobacillus casei* (LC) suppressed subcutaneous tumor growth in BALB*/c* mice. (**a**) Schematic representation of the in vivo tumor model. Female BALB*/c* mice received daily 10^9^ CFU of live *L. casei per os* for 13 days (*n* = 9/10). Control group received PBS (*n* = 9/10). On day 10, 5 × 10^6^ CT26 cells were injected subcutaneously. Growing tumors were excised 7 days post-inoculation of CT26 cells; (**b**) Representative photographic presentation of tumors from both groups; (**c**) Diagram showing mean tumor volume inhibition in probiotic-fed mice (LC) compared to control mice. *, *p* < 0.05, groups were repeated three times.

The protective effect of *L. casei* against CT26-induced colon carcinoma was evaluated. As shown in Figure 1a, BALB*/c* mice received a daily oral administration of 10^9^ colony-forming unit (CFU) live bacteria for 13 days. Control mice received an equal volume of phosphate-buffered saline (PBS). At day 10, 5 × 10^6^ CT26 cells were injected subcutaneously. Seven days post CT26 inoculation, animals were euthanized by cervical dislocation and tumors were isolated. No signs of disease or discomfort were observed during the experimental procedure. Oral *Lactobacillus* administration resulted in a statistically significant tumor growth inhibition compared to control mice that only received PBS for the same time period (Figure 1b). The respective box-plots of each group of mice are illustrated in Figure 1c. Notably, the tumor volume was significantly suppressed in the *L. casei* administered BALB/*c* mice by 63.1% compared to the control group (*p* < 0.05).

### 2.2. Elevated IFN-γ and IL-12 Production in Response to Lactobacillus casei Administration

**Figure 2:**
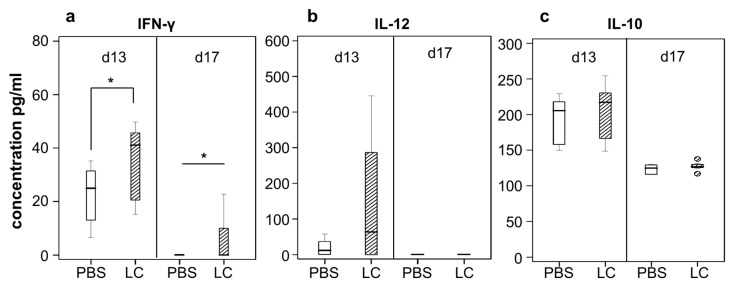
Effect of *Lactobacillus casei* administration on the ex vivo cytokine production. BALB*/c* mice received daily 10^9^ CFU of live *L. casei* for 13 days (*n* = 4/5). Control group received PBS (*n* = 4/5). On day ten (10) of oral administration, 5 × 10^6^ CT26 cells were injected subcutaneously. PP were excised after euthanasia three (d13 from start) or seven (d17 frorm start) days post-inoculation of CT26 cells. Cytokine levels of (**a**) IFN-γ, (**b**) IL-12 and (**c**) IL-10 were quantified by ELISA in a PP lysate of *L. casei* (LC)-fed mice as compared to the respective lysate of PBS-fed control mice. Cytokine production was determined at the indicated time points by ELISA following the manufacturer’s instructions. *, *p* < 0.05, groups were repeated two times.

In order to examine the effect of lactobacilli administration in intestinal cytokine secretion, PP isolated from mice were mechanically homogenized in PBS and cytokine production was evaluated with enzyme-linked immunosorbent assay (ELISA ) (Figure 2). A statistically significant increase in IFN-γ was observed in patches originating from treated mice both three (3) and seven (7) days (*p* < 0.05) post CT26 inoculation, at d13 and d17 from start, respectively (Figure 2a). IL-12 levels were also found to be elevated in treated mice on day 13, while levels of IL-12 were undetectable 7 days post cancer cell injection (Figure 2b). No significant differences in IL-10 levels were detected between the two groups (Figure 2c). It is worth mentioning that production of all three cytokines examined was elevated at three days post CT26 inoculation, which corresponds to the pause of probiotic administration (d13), while IL-10 was the only one detected in the patches of control animals at the endpoint of the experimental procedure, 7 days after tumor establishment (d17).

### 2.3. Lactobacillus casei Consumption Shows Distinct Immune Effector Cells in the Spleen

**Figure 3:**
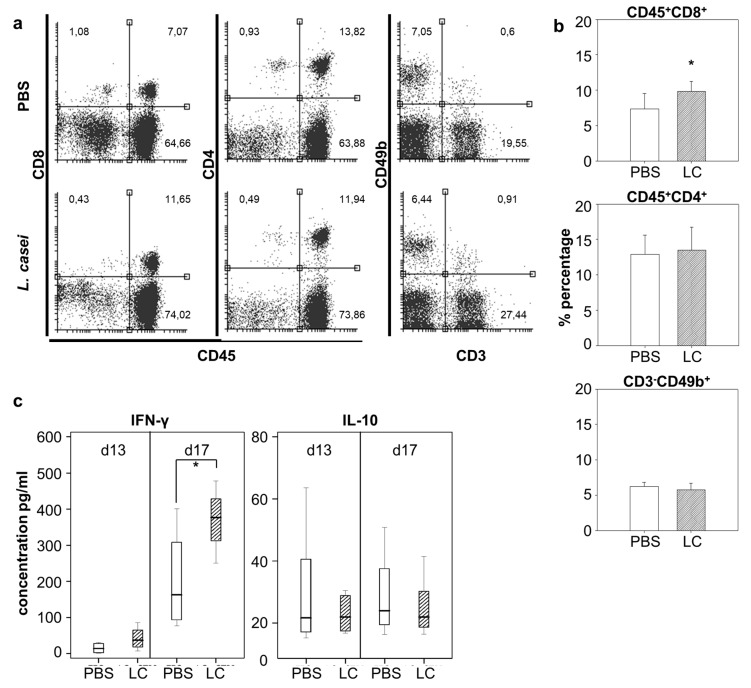
Effect of *Lactobacillus casei* administration on the percentage of effector immune cells in the spleen. BALB*/c* mice received daily 10^9^ CFU of live *L. casei per os* for 13 days (*n* = 4/5). Control group received PBS (*n* = 4/5). On day ten (10) of oral administration, 5 × 10^6^ CT26 cells were injected subcutaneously. Spleen cells were isolated from mice three (d13 from start) or seven (d17 from start) days post CT26 injection. Cells isolated at d17 were analyzed with flow cytometry. Isolated cells from both time points were co-cultured ex vivo with 10^8^ CFU/mL of *L. casei* for 48 h. (**a**) Representative dot plots for each subtype of immune cells (CD8^+^ T cells, CD4^+^ T cells, NK cells) in the spleen; (**b**) Frequencies of immune cell subtypes present in the spleen; (**c**) Cytokine production of spleen cells isolated from probiotic-fed mice at d13 or d17. *, *p* < 0.05, groups were repeated two times.

We then aimed to observe the effect of *L. casei* administration on selected lymphocyte populations in the spleen. Harvested spleens were homogenized, and following erythrocyte depletion, single-cell spleen cells were labeled with appropriate antibodies and analyzed with flow cytometry. As shown in Figure 3a,b, analysis of spleen cells unveiled a 2.5% increase (*p* < 0.05) of CD45^+^CD8^+^ T cells in probiotic-fed mice as compared to control animals, while there were no distinguishable differences on CD4^+^ T cells or CD3^-^CD49b^+^ NK cell populations between the two groups. Cytokine secretion from cultured spleen cells was evaluated by ELISA. Re-stimulation of splenocytes with *L. casei* for 48 h in vitro revealed an increase in IFN-γ secretion in the culture medium, an effect more prominent (*p* < 0.05) with spleen cells isolated at d17, seven (7) days post cancer cell injection. Production of IL-10 after *L. casei* re-stimulation in vitro was demonstrably not affected by consumption of the probiotic at either time point of spleen cell isolation (Figure 3c).

### 2.4. Lactobacillus casei Administration Resulted in Distinct Immunoadjuvant and Pro-apoptotic Effects in the Tumor

**Figure 4:**
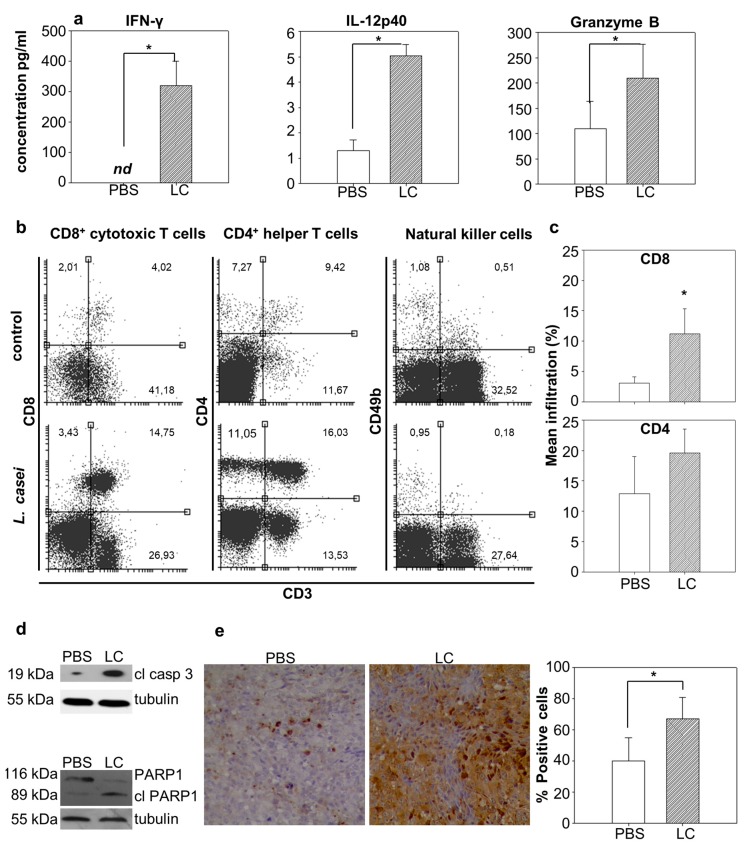
Dietary oral administration of probiotic *Lactobacillus casei* (LC) resulted in distinct immunoadjuvant and pro-apoptotic activities in tumor-bearing mice. Excised tumors from tumor-bearing LC-treated or control (PBS-treated) mice (*n* = 6) were either fixed in formalin, cut in sections, stained and observed under a microscope or mechanically homogenized. Homogenized tumors were further treated for protein isolation and analysis with ELISA and western blot, or enzymatically digested for cell isolation and flow cytometry analysis. (**a**) Cytokine production (IFN-γ, IL-12p40, Granzyme B) in the tumor tissue excised from treated animals as compared to tumor tissue from control mice; (**b**) Representative dot plots showing the percentage of each subtype in TILs; (**c**) Diagram of the frequency of TILs as percentage of mean infiltration; (**d**) Western blot analysis for the cleavage of caspase 3 and PARP1. Please note the increase in cleaved caspase 3 and cleaved PARP1 in LC-treated mice as compared to PBS-treated mice; (**e**) Representative images of tumor sections from control and LC-treated mice, stained for cleaved caspase 3. Please note the increase in the percentage of positive cells for caspase 3 activation in sections originating from animals that received oral administration of live *L. casei.* *, *p* < 0.05, groups were repeated three times.

We then wanted to examine the effect of *L. casei* administration in the tumor microenvironment, and the immune mechanisms that promoted tumor growth inhibition. The production of the cytokines IFN-γ, IL-12p40 and granzyme B was analyzed by ELISA in homogenized excised tumors from *L. casei*-fed as compared to PBS-fed mice. Increased IFN-γ production (*p* < 0.05), raising from undetectable levels in control animals to 319.8 ± 81.6 pg/mL in treated animals, was detected. Levels of IL-12p40 were slightly (from 1.3 ± 0.42 pg/mL to 5 ± 0.46 pg/mL) but significantly (*p* < 0.05) increased in tumors of mice that received the microorganism. A two-fold increase in Granzyme B accumulation was observed; Granzyme B production in the tumor microenvironment was increased (from 109.4 ± 54.7 pg/mL to 210.1 ± 67.3 pg/mL) in mice receiving lactobacilli (Figure 4a). Moreover, targeted migration and infiltration of immune cells in the tumor was evaluated by flow cytometry. Freshly isolated tumors were mechanically homogenized and then enzymatically digested to obtain single cell suspension. Subsequently, tumor cells were stained with appropriate antibodies. Analysis of tumor immunophenotype revealed a significant increase in tumor-infiltrating lymphocytes (TILs) (Figure 4b,c). The effect was more prominent in the case of CD3^+^CD8^+^ cytotoxic T cells that demonstrated an almost four-fold (*p* < 0.05) increase in treated probiotic-fed animals. CD3^+^CD8^+^ cytotoxic T cell infiltration ranged from 3.12 ± 0.97% in control animals to 11.17 ± 4.09% in lactobacilli-administered mice. CD3^+^CD4^+^ helper T cells did also accumulate in the tumors of treated animals although this difference was not statistically significant. There was no significant infiltration of NK cells in the tumor tissue in control or treated mice.

Cancer cell apoptosis was evaluated in the tumor by detection of characteristic apoptotic markers by Western blot analysis and immunohistochemistry. Tumor lysate was prepared as mentioned above for cytokine analysis and levels of cleaved caspase 3 were evaluated by Western blot. Caspase 3 was significantly active in the tumor of mice that were fed with probiotic bacteria, as levels of the cleaved, active fragment of caspase 3 were approximately 2.5 times higher than those observed in animals from the control group (Figure 4d, Supplementary Figure S1). Immunohistochemical analysis of tumor sections corroborated this observation, as there was an approximately 20% increase in the number of cells positive for cleaved caspase 3 in the tumors of treated animals (Figure 4e). Successful induction of apoptosis in tumor cells was estimated with Western blot for PARP1, a typical apoptotic marker. The cleavage of PARP1 with a corresponding fragment of 89 kDa, by activated caspase 3 among other molecules, is a hallmark of the apoptotic pathway execution. As shown in Figure 4d, cleavage of PARP1 was more prominent in tumor extracts of mice receiving *L. casei* compared to control mice, as evidenced by a decrease in the levels of the full-length protein at 116kDa and subsequent increase in the intensity of the cleaved fragment at 89 kDa.

### 2.5. Probiotic Administration Resulted in Altered Interleukin and Chemokine Production in the Tumor

**Figure 5:**
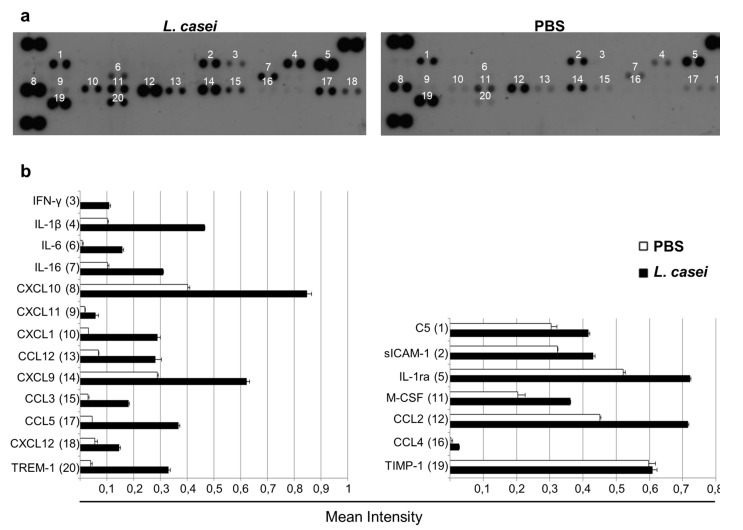
Administration of probiotic *Lactobacillus casei* (LC) resulted in increased production of various interleukins and chemotactic factors in the tumor. Tumors were homogenized, three tumors per group were pooled, and then analyzed for protein expression with a commercially available proteome profiler array. (**a)** Images of dot plots. Duplicate dots represent a protein expressed in tumor tissues of LC-fed or PBS-fed mice; (**b**) Both plots were scanned and signal intensity, normalized against positive controls provided by the manufacturer, was determined. Note the elevated levels of IFN-γ that was previously detected, and the accumulation of interleukins, like IL-1b and IL-16 and chemotactic agents such as CXCL9, CXCL10 and CXCL11 as well as CCL3, CCL4 and CCL5.

As immunomodulation in response to *L. casei* administration was strongly suggested by the aforementioned results, we decided to further investigate cytokine accumulation and chemokine production in the tumor. Tumor lysate was further analyzed for the production of 40 different cytokines with a proteome profiler array. As shown in Figure 5, this analysis verified the aforementioned results for IFN-γ, and also revealed increased production of various other pro-inflammatory interleukins, such as IL-1b and its corresponding receptor, and IL-16. Interestingly, a more prominent effect was revealed concerning various chemotactic agents. Of note is the accumulation of ligands for the C-C chemokine receptor 5 (CCR5) and C-X-C chemokine receptor 3 (CXCR3) receptors such as chemokine (C-C motif) ligand 3 (CCL3), CCL4, CCL5 and C-X-C motif chemokine ligand 9 (CXCL9), CXCL10, CXCL11, respectively, that act as signals for the migration of CD8^+^ T cells and NK cells and are associated with IFN-γ and anti-tumor effects. These data show that *L. casei* administration induces potent Th1 immune responses.

## 3. Discussion

*Lactobacillus casei* ATCC393 is an established probiotic strain used in fermented dairy products and functional foods [32,33,34,35]. We have previously demonstrated that it exhibits anti-proliferative and pro-apoptotic effects against colon carcinoma [11]. Here, we investigated its immunomodulatory activity as a potential mechanism mediating those effects.

Various probiotic lactic acid bacteria have been attributed with anti-tumor activity [36,37], although primarily in pre-clinical animal models. In our experiments, we observed that prophylactic administration of live *Lactobacillus casei* reduced CT26 tumor volume by approximately 63%, in a syngeneic mouse tumor model. As accumulation of probiotic microorganisms and interactions with the underlying immune cells in PP is one of the routes by which probiotics are processed [38], we examined cytokine production in this region. Levels of IFN-γ and IL-12 were significantly elevated in *L. casei*-fed mice at the endpoint of the probiotic administration (d13 from start) 3 days post CT26 inoculation in accordance with a study by Chiba et al [18], and increased IFN-γ production in PP was persistent at the end of the experimental procedure, 7 days after tumor establishment. In order to clarify whether this modulation was systemic, cytokine secretion was also quantified in in vitro cultures of spleen cells, stimulated with *L. casei.* Higher levels of secreted IFN-γ were observed when spleen cells, isolated from mice three (d13 from start) or seven (d17 from start) days post tumor cell inoculation, were cultured in vitro in the presence of 10^8^ CFU/mL of *L. casei* for 48 h. The immunosuppressive regulatory cytokine IL-10 was not significantly affected by the consumption of the probiotic as levels of this interleukin were neither altered in PP nor spleen cell culture at any time point examined.

Both IFN-γ and IL-12 are associated with enhanced anti-tumor activity [39,40]. IL-12 in particular induces proliferation of NK and T cells, as well as production of cytokines involved in cytotoxicity, including IFN-γ. It is also an essential cytokine for Th1 differentiation, which is required for the generation of T-cell mediated immunity to cancer [41]. IFN-γ is an effector molecule with a variety of pleiotropic effects. It acts through a positive feedback loop to stabilize and promote Th1 lineage of helper cells and innate immunity populations, and simultaneously inhibits Th2 differentiation and IL-4 production. Moreover, it induces elevated expression of major histocompatibility complex (MHC) class I and II molecules and transcription factors, and the majority of antigen processing and presentation mechanisms. Apart from interacting with immune cells, IFN-γ also targets tumor cells. Results of this interaction include upregulated expression of caspases, CD95 (Fas) and secretion of CD95 ligand (Fas ligand), and TNF-related apoptosis-inducing ligand (TRAIL), and enhanced detection of tumor cells by CD8^+^ and CD4^+^ T cells. By acting on both ends of the antigen presentation process, it ensures an effective tumor eliminating response [42].

As evidenced above, a critical step in cancer cell cytotoxicity is the accumulation of the signaling molecules and effector cells in the tumor. In the current study, the observed increased production of IFN-γ and IL-12 was persistent in the tumor microenvironment, following live *L. casei* administration. Moreover, examination of cytokine/chemokine production in the tumor microenvironment revealed an increase in the levels of chemoattractive cytokines such as CCL3, CCL4, CCL5, CXCL9, CXCL10 and CXCL11. These are ligands for the receptors CCR5 and CXCR3, respectively, that have been shown to be over-expressed in response to IFN-γ [43] and IL-12, and facilitate migration of activated cytotoxic T cells (CTLs) and NK cells in the tumor microenvironment [44]. Ligands of the CXCR3 receptor and especially CXCL10 are exceptionally important in tumor eradication, as they are not only highly effective in attracting cytotoxic populations in the tumor [45], but have also been linked with prevention of angiogenesis, a significant milestone of tumor establishment, by inhibiting the proliferation of endothelial cells [46]. Tumor infiltration by CD8^+^ T cells is associated with a better prognosis in most tumor types including colorectal cancer [47,48]. As shown above, *L. casei* administration led to a slight enrichment of the CD8^+^ T cell population in the spleen and more importantly in increased tumor infiltration of the aforementioned CD8^+^ T cells. While both populations were increased in probiotic fed-mice, the effect was more prominent in tumor infiltrated CTLs, where the number of CD8^+^ T cells was 3.5 times higher in mice receiving probiotics, compared to a much more subtle increase in the spleen. This observation corresponds with the secretion of chemoattractants in the tumor. As shown by Allen et al. [49], CCL3 secretion in the tumor results in higher IFN-γ production and as mentioned before, IFN-γ further enhances accumulation of molecules contributing to targeted migration and tumor specific cytotoxicity, such as CXCL9, CXCL10 and CXCL11 [50], especially in combination with CCL5 [51], which was also potently increased in our experiments. The combination of IL-12, IFN-γ, CXCL9 and CXCL10 has been shown to be strongly correlated with recruitment of CD8^+^ T cells in the tumor, in colorectal [52] and lung cancers [53]. A prominent mechanism of CTLs involved in cancer cell destruction is the direct exocytosis of perforin and granzyme B containing granules into the target cell [54,55]. Perforin forms pores in the plasma membrane of the target cell that act as ways of delivery of granzyme B to it [56]. Therefore, we quantified granzyme B production in the tumor as a means of examining the successful cytotoxic activity of CTLs and we observed a notable increase in tumors of probiotic-fed mice, where levels of granzyme B approximately doubled. Secretion of CXCL10 and CCL5 in the tumor has been associated with accumulation of granzyme producing CD8^+^ and IFN-γ producing CD4^+^ T cells in colorectal carcinomas, and early TNM staging [43]. The desired final outcome of the accumulation of cytotoxic CD8^+^ T cells, IFN-γ and granzyme B in the tumor is the destruction of cancer cells, most commonly by the induction of the apoptotic mechanism in the tumor cells [56]. To verify if this was indeed the case in our experiments, we investigated the activation of the most prominent effector caspase 3, which is the endpoint of all apoptotic pathways. We observed an increase in the number of cancer cells positive for the presence of active cleaved caspase 3 in tumor sections, as well as elevated levels of the cleaved fragment in tumor lysates of mice that consumed lactic acid bacteria. We also examined the characteristic apoptotic marker PARP1, since cleavage and inactivation of the full-length protein is a common indicator of apoptosis. As expected, levels of the full-length protein were higher in tumor extracts of control animals receiving PBS, while increased cleavage of PARP1 was observed in tumors of treated mice.

Secretion of IFN-γ and cytotoxic granules are two distinct tasks performed by effector CTLs, specific for tumor-associated antigens that directly interact with malignant cells, during tumor cell eradication [57]. In conclusion, our results suggest an immunomodulatory effect of preemptive oral administration of *L. casei* in an in vivo syngeneic colon cancer model. These results should be carefully considered for transfer to humans as there are limitations in mouse models regarding translation of the findings from preclinical studies to clinical application. Consumption of the probiotic was shown to impair CT26 tumor growth, an effect that could be attributed to the production of immunostimulatory cytokines in lymphatic organs and the secretion of chemoattractant molecules, associated with migration of CTLs in the tumor. The end point of the aforementioned modulation in the cytokine production profile is enhanced tumor infiltration by T cells, and especially CD8^+^ CTLs, an event that is essential and plays a crucial role in cancer immunotherapy [58].

## 4. Materials and Methods 

### 4.1. Animals and Ethics Statement

Female BALB/*c* mice were raised in the Animal House unit (Laboratory of Experimental Surgery and Surgical Research at Democritus University of Thrace, Alexandroupolis, Greece). Two groups of 10 mice (6-8 weeks old, weight 20–25 g) were used in each experiment. Animals were housed in polycarbonated cages at room temperature and were provided with commercial food and tap water ad libitum.

Animal experiments were approved by the Animal Care and Use Committee of the Veterinary Department of Evros Prefecture (license number 4766/28-3-2013) since it complied with the requirements set by Directive 86/609/EEC and PD 160/91. All animal experiments were conducted in accordance with the 3 R’s (replacement, refinement, reduction).

### 4.2. Cell Lines and Bacterial Culture Conditions

CT26 colon cancer cells were routinely cultured under sterile conditions in standard Dulbecco’s Modified Eagle Medium (DMEM) (Life Technologies, Carlsbad, CA, USA) supplemented with 10% heat inactivated fetal bovine serum, 2 mM glutamine, 100 μg/mL penicillin and 100 ug/mL streptomycin (Life Technologies, Carlsbad, CA, USA) at 37 ·C in a humidified incubator (5% CO_2_).

*Lactobacillus casei* ATCC 393 (DSMZ, Braunschweig, Germany) was grown at 37 ·C in De Man, Rogosa and Sharpe (MRS) Broth without agitation and harvested in late log phase (10^9^ CFU/Ml). Bacteria were collected by centrifugation at 1700× *g* for 15 min at 4 ·C, washed in PBS and resuspended in PBS to the desired final concentration of 10^9^ CFU/150 μL for in vivo experiments. For the in vitro co-incubation experiments, *L. casei* was resuspended in RPMI (Life Technologies, Carlsbad, CA, USA) medium supplemented with 10% heat inactivated fetal bovine serum, 2 mM glutamine and 100 U/mL penicillin, 100 μg/mL streptomycin and 50 μg/mL gentamycin (Life Technologies, Carlsbad, CA, USA).

### 4.3. CT26 Syngeneic Tumor Model and Lactobacillus casei Administration

The experimental protocol was approved by the Animal Care and Use Committee of the local Veterinary Service and was in compliance with Directive 86/609/EEC. Female BALB/*c* mice (6–8 weeks old, weight 20–25 g) were separated into two independent groups. The group of *L. casei* (LC)-treated mice received *per os* for 13 days a daily dose of 10^9^ CFU/mL live *L. casei* suspended in 150 μL PBS. The group of control mice received an equal volume of PBS. At day 10, 5 × 10^6^ CT26 cells per mouse were injected subcutaneously. Mice were euthanized by cervical dislocation at two time points; three days post CT26 inoculation, corresponding with discontinuation of probiotic administration, and seven days post CT26 inoculation at full tumor development. Tumors, PP and spleens were isolated. Tumor incidence and tumor volume were determined. Tumor volume was calculated using the modified ellipsoid formula: (width^2^ × length)/2. During the course of the experiments, mice were monitored for signs of disease or discomfort.

### 4.4. Cytokine Analysis

Cytokine production (IL-10, IL-12p70 and IFN-γ) from PP and spleen cells as well as the secretion of Granzyme B, IFN-γ and IL-12p40 in the tumor were detected by ELISA (eBioscience, San Diego, CA, USA). Directly after euthanasia of mice, PP were isolated from small intestine as well as tumors. Both were mechanically homogenized in PBS supplemented with protease inhibitors (10 ug/mL Aprotinin, 10 ug/mL Leupeptin and 10 ug/mL Pepstatin). After homogenization, Triton X-100 (Applichem, Darmstadt, Germany) was added to a final concentration of 1%. Samples were frozen at −80 ·C, thawed and centrifuged for 5 min at 10,000× *g* to remove cellular debris. Spleens were collected from mice, homogenized in PBS containing 50 ug/mL gentamycin, centrifuged at 500× *g* for 5 min and washed once with PBS. Erythrocytes were depleted using 10× red blood cell (RBC) Lysis Buffer (eBioscience, San Diego, CA, USA). Briefly, cell pellet was suspended in 5 mL RBC Lysis Buffer and incubated for 5 min at room temperature. Subsequently, suspensions were centrifuged for 5 min at 500× *g* and washed once with PBS. Cells were then suspended in RPMI medium and splenocytes were treated with 10^8^ CFU/mL live *L. casei* for 48 h. PP, the supernatant of the tumor homogenate and the culture supernatant of spleen cells were examined for cytokine production by ELISA, according to the manufacturer’s instructions. Tumor lysate was further analyzed for the presence of 40 different cytokines using the Mouse Cytokine Array Panel A (ARY006, RnD Systems, Minneapolis, MN, USA), according to the manufacturer’s instructions. Homogenates from three tumors per group were pooled for this analysis. Signal intensity was calculated using ImageJ.

### 4.5. Flow Cytometry

Spleen cell suspension was prepared as mentioned above for cytokine analysis. TILs were isolated from freshly dissected tumors. Briefly, tumors were washed with PBS, minced using a razor blade and incubated with collagenase IV (Sigma Aldrich, St Louis, MI, USA, 0.5 mg/mL in RPMI) for 1 h at 37 ·C. Digested tissues were then filtered through a 100 μm pore size strainer to obtain a single-cell suspension and cells were washed once before staining. Approximately 10^6^ cells were suspended in PBS and incubated with CD16/CD32 Fc block antibody (Cat553141, BD Biosciences, San Jose, CA, USA) for 20 min at 4 ·C. Cells were washed with PBS and incubated with 1 μL of the appropriate fluorochrome-conjugated antibody for 40 min at 4 ·C. Cells were washed with PBS and incubation with conjugated antibodies was performed. Antibodies used in these analyses were from BD Biosciences (San Jose, CA, USA): FITC-CD45 (Cat 553772), FITC-CD8a (Cat 553031), PE-CD8a (Cat 553033), PE-CD4 (Cat 553730), PE-CD3e (Cat 553063), APC-CD4 (Cat 353051) and APC-CD49b (Cat 560628). Finally, cells were washed twice with PBS before flow cytometry analysis (Calibur, BD Biosciences, San Jose, CA, USA).

### 4.6. Immunohistochemistry

Extracted tumors were fixed in 10% formalin and then dehydrated in graded concentrations of ethanol, xylole and finally embedded in paraffin. Serial sections, 3 μm thick, from the formalin fixed, paraffin embedded tissue blocks were prepared and floated onto glass slides. A hemotoxylin and eosin stained section was obtained from each tissue block. All sections were deparaffinized and hydrated using graded concentrations of ethanol to deionized water. Tissue sections were subjected to quenching of endogenous peroxidase and antigen retrieval using microwaving in high pH citrate buffer. The primary antibody, cleaved caspase 3 (Cell Signaling, Danvers, MA, USA), was then applied to the tissues at a dilution of 1:1000 and left overnight. Bound antibody was then visualized with DAB chromogen, followed by counterstaining with hematoxylin. Negative controls were incubated and consisted of the same immunohistochemical method with omission of the primary antibody. An image analysis system composed of the Olympus BX43 upright microscope, digital camera Olympus Cam-SC30 (Olympus Europa, Hamburg, Germany) and soft analysis (analySISH) was used in the tumor sections (stained with antibodies and counterstained with hematoxylin). A continuous score system was adopted by using the ×40 objective lens and counting at least 10 fields selected on the basis that they contained immunopositive tumor cells. The number of immunopositive cells was divided by the total number of the counted cells, and the expression was defined as the percentage of positive cells in the total number of the counted cells. The score was performed by evaluation of the staining by two independent observers using the light microscope.

### 4.7. Western Blot Analysis

Apoptosis induction in the tumor was evaluated by detection of cleaved fragments of the effector caspase 3 and the characteristic apoptotic marker PARP1 by Western immunoblot. Tumor lysate was prepared as described above for cytokine analysis. Protein concentration of samples was determined by the bicinchoninic acid (BCA) protein assay kit (Thermo Scientific, Waltham, MA, USA). Standard Western blot analysis was performed for the detection of cleaved caspase 3 as well as whole and cleaved PARP1. Equal amounts of protein extracts were loaded onto sodium dodecyl sulfate (SDS) polyacrylamide gels. A 12% polyacrylamide gel with subsequent blotting onto 0.2 μm polyvinylidene difluoride (PVDF) membrane (Immobilon, Darmstadt, Germany) was used for caspase 3, while 10% polyacrylamide gel and 0.45 μm PVDF membrane (Amersham, Little Chalfont, United Kingdom) were used for PARP1. Blocking of non-specific sites was done in 5% non-fat milk and the membrane was incubated overnight with primary antibodies from Cell Signalling (Danvers, MA, USA; cleaved caspase 3-9664, 1:500; PARP1-9532, 1:1000) in blocking buffer at 4 ·C, and with the horseradish peroxidase (HRP) conjugated anti-rabbit secondary antibody (1:5000) in blocking buffer for 1 hour at room temperature. The protein band was visualized by autoradiography using ECL HRP chemiluminescent substrate (Life Technologies, Carlsbad, CA, USA) and exposing to Kodak film. An anti-beta-tubulin antibody (1:20,000, Sigma Aldrich, St Louis, MI, USA), accompanied by HRP-conjugated anti-mouse secondary antibody (1:5000) was used as a loading control. Intact PARP1, cleaved PARP1, cleaved caspase 3 and beta-tubulin were detected at 116 kDa, 89 kDa, 19 kDa and 55 kDa, respectively, as determined by monitoring the molecular weight with a pre-stained protein marker (Nippon Genetics, Tokyo, Japan).

### 4.8. Statistical Evaluation

Data were analyzed with the IBM SPSS Statistics 22 software (IBM, Armonk, NY, USA). Statistical differences for independent samples were analyzed by performing Student’s t-test for parametric and Mann-Whitney test for non-parametric variables. Differences were considered significant when *p* < 0.05. A minimum power of 0.80 was used when calculating sample size.

## 5. Conclusions

The present study investigated the role of dietary probiotic bacteria and host anti-cancer immune responses raised against experimental colon carcinoma in mice. The aim was to understand how oral administration of a single probiotic bacterium affects local and systemic immune responses towards syngeneic tumors. Our results suggest an immunomodulatory effect of preemptive oral administration of *L. casei* in the in vivo syngeneic CT26 cancer model. Daily consumption of the probiotic *Lactobacillus casei* strain was shown to impair tumor growth, an effect that could be attributed to the production of Th1 immunostimulatory cytokines and secretion of chemoattractant molecules in tumor tissue, accompanied by a migration of cytotoxic T cells in the tumor. The end point of the aforementioned modulation in the cytokine production profile was enhanced tumor infiltration by T cells, and especially CD8^+^ CTLs, an important event with crucial role in cancer immunotherapy. Our results suggest that cocktails of beneficial bacteria may be used as novel anticancer immunoadjuvant agents in future therapeutic regimens. However, due to limitations of pre-clinical mouse models, caution should be taken when interpreting these findings for clinical application; further work in that direction is needed. Ongoing research in this field will ultimately lead to a better understanding of the role of diet and probiotics in immune function and diseases, facilitating thereby the use of beneficial probiotic supplements to improve human health. 

## Supplementary Materials

Supplementary materials can be found at https://www.mdpi.com/2072-6694/12/2/368/s1.
