# Supplementary material for: Immune Responses Raised in an Experimental Colon Carcinoma Model Following Oral Administration of Lactobacillus casei
Source: Cancers (Basel). 2020 Feb 5;12(2):368. doi: 10.3390/cancers12020368 (PMC7072577; doi:10.3390/cancers12020368)
Supplement: Supplementary file 1 [file cancers-12-00368-s001.zip › Supplementary Figure legend S1.docx]

**Figure S1.** Oral administration of *Lactobacillus casei* (LC) resulted in enhanced apoptotic activity in the tumour. Normalized pixel intensity of cleaved caspase 3, PARP1 and cleaved PARP1 with b-tubulin. Bars are means with standard deviation from tumour pairs (PBS, LC) from independent experiments. Elevated levels of caspase 3 activation and PARP1 inactivation by cleavage were detected in mice receiving the probiotic
