# Supplementary figures and images for: Immune Responses Raised in an Experimental Colon Carcinoma Model Following Oral Administration of Lactobacillus casei
Source: Cancers (Basel). 2020 Feb 5;12(2):368. doi: 10.3390/cancers12020368 (PMC7072577; doi:10.3390/cancers12020368)

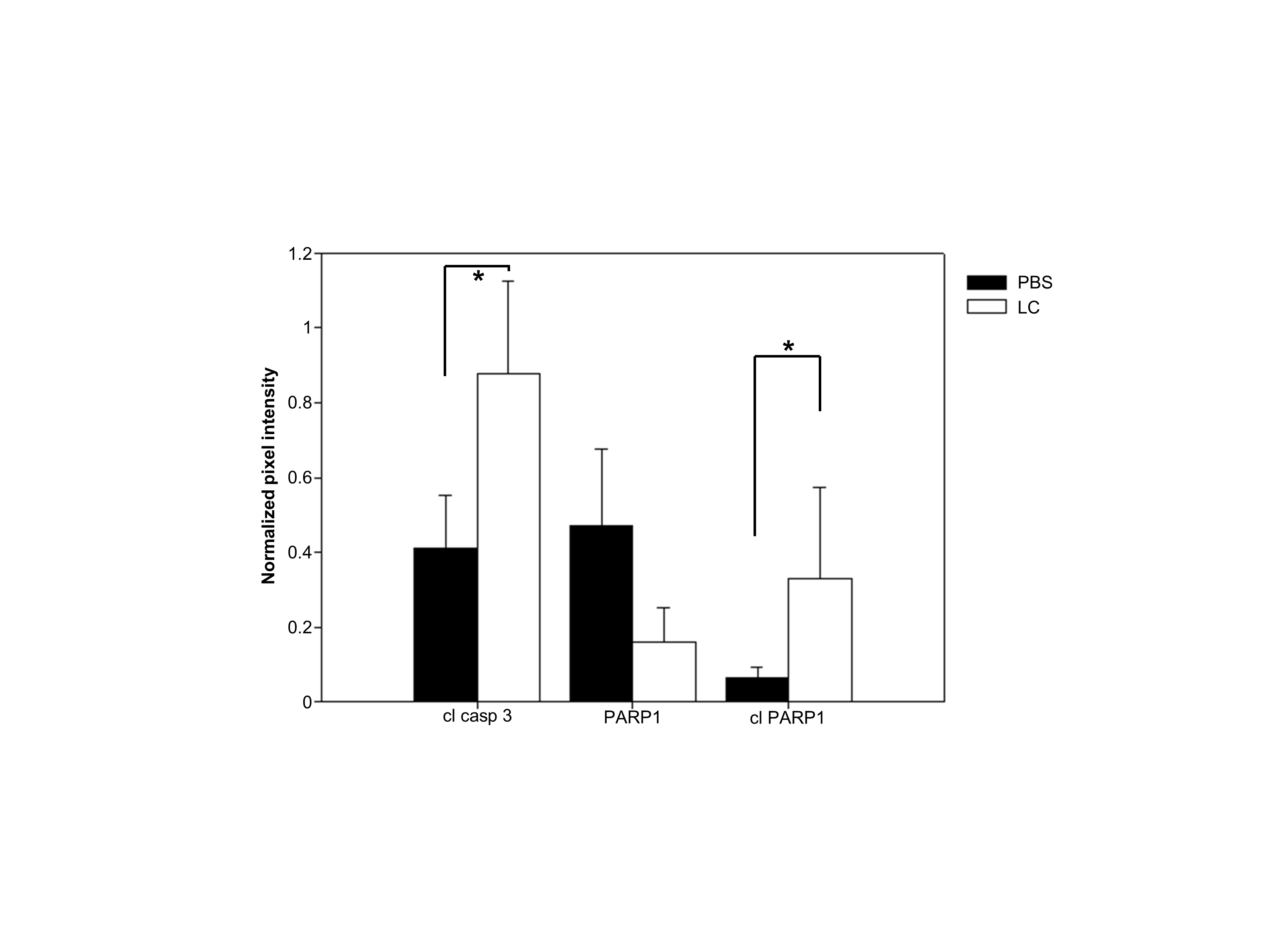

Supplement: Supplementary file 1 [file cancers-12-00368-s001.zip › Supplementary Figure S1.tif]
